# Editorial: Advances in therapeutic strategies of inborn errors of immunity

**DOI:** 10.3389/fimmu.2023.1328846

**Published:** 2023-11-09

**Authors:** Rakesh Kumar Pilania, Taru Goyal, Surjit Singh

**Affiliations:** Pediatric Allergy Immunology Unit, Advanced Pediatrics Centre, Postgraduate Institute of Medical Education and Research, Chandigarh, India

**Keywords:** inborn errors of immunity, treatment, therapeutic strategies, advances, primary immunodeficiencies

Inborn Errors of Immunity (IEIs), hitherto known as primary immunodeficiency disorders, represent a diverse group of genetic conditions characterized by impaired immune functions. Clinical manifestations can be variable and affected individuals may present with recurrent infections, autoimmunity, and other immune-related complications. In recent years, there has been remarkable progress in the field of IEIs (1, 2). These breakthroughs have paved the way for the development of innovative therapeutic strategies, offering hope for significant improvements in the quality of life of affected individuals.

The Research Topic entitled, ‘*Advances in Therapeutic Strategies of Inborn Errors of Immunity*,’ highlights promising approaches that are revolutionizing the management landscape of IEIs. Eight manuscripts (4 review articles and four clinical reports) have been included in this Research Topic.

Hematopoietic stem cell transplant (HSCT) is now the standard of care for the treatment of several IEIs (3). Despite major advances in HSCT for IEIs, several challenges still remain. These include, amongst others, issues related to donor selection and the potential risk of post-HSCT complications (e.g., graft versus host disease). Gene editing, which involves specially designed nucleases, introduces stable genetic alterations at a specific location. This technology has been recently introduced to overcome the potential barriers in treating IEIs (4, 5). Several programmable nucleases have been used for gene editing – these include zinc-finger nucleases (ZFN), transcription activator-like effector nucleases (TALEN), and Clustered Regularly Interspaced Short Palindromic Repeats/CRISPR-associated protein (CRISPR/Cas). CRISPR/Cas is the most widely used gene editing tool (6).

Pinto and Neves, report the application of precision medicine in the treatment of IEIs which allows accurate diagnosis and use of targeted treatment at a specific defect. They have also discussed the role of HSCT, gene therapy, and biologics or molecules targeting specific immune pathways and cell functions. Similarly, Meng et al. have reviewed recent advances in diverse genome editing technologies, their applications, and the associated challenges in developing and implementing these treatment modalities in managing patients with IEIs. The ClinVar database reveals that pathogenic variants related to IEIs may include SNVs, deletions, and insertions. The authors have also shown that CRISPR/Cas9-based genome editing technologies have significantly impacted the treatment algorithms in IEIs. Additionally, newer tools such as base editing (BE) and prime editing (PE) platforms that have recently emerged may also have the potential for treating IEIs. Liu et al., in the same issue, have reviewed the role of CRISPR/Cas gene editing technology in the management of IEIs. The authors have outlined the development and progression of CRISPR/Cas gene-editing systems, including breakthroughs like double-strand break (DSB)-based gene editing and DSB-free base editing or prime editing systems. They have reviewed the preclinical studies and ongoing clinical trials using this technology in IEIs (7). To summarize, recent advancements in the curative management of IEIs have revolutionized the available treatment options. However, more research is required to address specific challenges, customize treatments based on mutations, optimize editing efficiency, and improve the engraftment and self-renewal abilities of gene-corrected hematopoietic stem cells for successful clinical outcomes. Additionally, owing to the rarity of IEIs, multi-center collaboration is crucial to gain valuable insights into the management and understanding of IEIs.

Mendelian susceptibility to mycobacterial disease (MSMD) comprises a heterogeneous group of disorders characterized by a selective predisposition to intracellular organisms due to defects in the IFN-γ/IL-12 signaling pathway (8). In a systematic review, Xia et al. report on 19 genes linked to 34 clinical phenotypes that have been unveiled in the last 26 years since the recognition of the first genetic etiology of MSMD in 1996. The authors have also systematically reviewed clinical and molecular profiles and outcomes of patients with MSMD reported from 20 studies in China. The authors enrolled 65 patients and identified variants in 9 genes (viz. *IFNGR1, IFN* ***γ*** *R2, ISG15, IL12β, IL12Rβ1, STAT1, TYK2, NEMO, CYBB)*. Based on Chinese literature, it seems that IL-12RB1 deficiency is the most common molecular defect, accounting for 52% of all cases; followed by IFN*γ*R1 (22%) and STAT1 (9%). Similar experiences have also been reported in a multicentric study from India wherein IL-12Rβ1 (46%) constitutes the most prevalent genetic form of MSMD followed by complete IFN-γR1 (14%), complete IFN-γR2 (14%), PD-IFN-γR1 (13%), and complete STAT1 defect (7%). A greater frequency of IFNγR2 defects has been observed in the Indian cohort as opposed to the Chinese cohort (9). Notably, 92.3% (60/65) of the Chinese MSMD patients developed BCG disease following routine BCG vaccination. Also, disseminated BCG-osis was the most common presenting manifestation (82%) in the Indian study as well (9).

The JAK/STAT signaling pathway plays a crucial role in immune functions, and several IEIs have been described owing to a defect in this pathway directly or indirectly. The association between the cytokines and the JAK-associated receptors leads to the stimulation of certain specific intracellular signals which in turn play a significant role in the pathophysiology of various immune diseases (10). Thus, JAK inhibitors represent a new class of small molecules that targets the JAK-STAT pathway. There have been several recent reports on the use of JAK inhibitors in patients with IEIs. Xie et al. report the use of ruxolitinib (JAK inhibitor) in a child with a gain-of-function (GOF) mutation in the STAT1 gene (c.854A>G, p.Q285R), resulting in a syndrome characterized by combined immunodeficiency, autoimmunity, and pure red cell aplasia (PRCA) in a previously suspected patient of lupus. In this case, the authors have used ruxolitinib as the preferred therapeutic option along with conventional therapies (viz. intravenous immunoglobulin, corticosteroids) to control the hyper-inflammation and hemolysis for bridging the HSCT. Niizato et al. report on the use of ruxolitinib in an infant with familial hemophagocytic lymphohistiocytosis (fHLH) type 3 who had uncontrolled cytokine storm despite initial therapy with dexamethasone and etoposide. The patient’s cytokine storm was effectively managed after ruxolitinib, allowing for successful HSCT. The report also reviews similar cases treated with ruxolitinib, underscoring positive outcomes in most cases. The authors have also demonstrated the utility of monitoring cytokine levels (e.g., interleukin-18, CXCL9, sIL-2R, and sTNF-RII) in fHLH patients as indicators of disease activity (11). These reports along with previously published literature support the potentially beneficial use of inhibition of the JAK-STAT pathway in patients with IEIs.

Severe combined immunodeficiency (SCID) is a heterogeneous group of monogenic defects characterized by profound T-cell deficiency. As the BCG vaccine is routinely administered at birth in several countries, a high percentage of SCID patients get vaccinated before their immune defect can be detected. This is particularly true in areas where newborn SCID screening programmes have still not been implemented (12, 13). Compared to other infants, these patients have a higher incidence of BCG disease after vaccination, and disease flares often occur post-HSCT. Liu et al. report the management of immune reconstitution inflammatory syndrome (IRIS) following HSCT in a 4-month-old boy with X-linked SCID in a setting of severe disseminated BCG infection. Despite receiving anti-tubercular therapy (ATT), the patient’s condition worsened, leading to multiple complications and the need to perform a non-conditioned allogeneic HSCT. After HSCT, the baby developed hepatic veno-occlusive disease, acute graft-versus-host disease, and IRIS. The baby was treated successfully with glucocorticoids, ibuprofen, and adjustments to immunosuppressive medications in addition to ATT. This case highlights the challenge of managing BCG vaccine-related nuisance in patients with SCID post-HSCT.

It has been 55 years since the first HSCT was carried out for a child with SCID. Over the years, there have been major advances in the timely diagnosis of IEIs, donor selection, and conditioning regimens (3). For patients with IEIs where HSCT is curative and an HLA-matched donor is unavailable, haploidentical HSCT represents an alternative approach. Literature on the success of haploidentical HSCT in patients with IEIs has evolved over the last two decades, and has shown steadily improving survival rates (14, 15). Yamashita et al. reported successful HLA-haploidentical HSCT in a 5-month-old girl with leukocyte adhesion deficiency type I (LAD-I) using post-transplant cyclophosphamide (PT-CY). LAD-I is a rare genetic disorder characterized by defective leukocyte adhesion and impaired migration, primarily caused by defects in the ITGB2 gene. Allogeneic HSCT is the only curative treatment for LAD-I. However, due to the unavailability of a suitable donor, haploidentical HSCT was performed, with the mother being the donor, and a successful complete chimerism was achieved. The patient experienced mild graft-versus-host disease, cytomegalovirus reactivation, and veno-occlusive disease/sinusoidal obstruction syndrome, which were effectively managed. With improved conditioning regimens, HLA-haploidentical HSCT is now a safe and effective mode of treatment for patients with IEIs where HLA-matched donors are unavailable.

Recent developments in curative therapies for IEIs represent a major advance in the treatment of these conditions. However, this is clearly work in progress and there are several challenges that still need to be overcome.

## Author contributions

RP: Data curation, Writing – original draft, Writing – review & editing. TG: Data curation, Writing – review & editing. SS: Conceptualization, Writing – review & editing.
